# A Rare Presentation of Clozapine-Induced DRESS Syndrome

**DOI:** 10.1155/2018/1346351

**Published:** 2018-06-11

**Authors:** Carmel Moazez, Yasir Rudha, Natasha Narang, Tina Younger

**Affiliations:** Maricopa Medical Center, 2601 E. Roosevelt St., Phoenix, AZ 85008, USA

## Abstract

Drug reaction with eosinophilia and systemic symptoms (DRESS syndrome) is an uncommon side effect of certain medications. It causes a skin reaction, with eosinophilia and other organ involvement. This case describes a presentation of a 32-year-old female with a past medical history significant for schizophrenia and bipolar disorder who presented for a rash. She had been started on clozapine 10 days prior to admission. After extensive workup she was found to have DRESS syndrome secondary to clozapine use. This is the second reported case in the literature of clozapine-induced DRESS syndrome. This case is unique because it is the first case to present with the common manifestations of DRESS syndrome including eosinophilia, rash, lymphadenopathy, and organ involvement after clozapine use.

## 1. Introduction

Drug reaction with eosinophilia and systemic symptoms (DRESS syndrome) is an uncommon side effect of certain medications. It causes a severe skin reaction and fever with eosinophilia and other organ involvement [1]. The incidence ranges from 1 in 1,000 to 1 in 10,000 drug exposures [2]. It is most commonly seen with use of allopurinol, sulfonamides, carbamazepine, and other anticonvulsants, but has been noted as a side effect of 44 drugs thus far [3]. This reaction is usually seen within 2–6 weeks of starting the offending agent and can be a life threatening reaction [1].

The most common organ systems that are involved in DRESS syndrome include lymphatic, hematologic, and hepatic systems. Other less commonly affected organs include the kidney, lungs, and heart. The typical rash seen in DRESS syndrome is a diffuse, pruritic, macular exanthema. The rash usually starts on the face, upper extremities, and upper trunk and then spreads to the lower extremities and lower trunk. DRESS syndrome will also present with facial edema in 25% of patients. Lymphadenopathy is a common finding and is seen in 75% of cases. There can be leukocytosis up to 50.0 × 10^9^, but initially patients might present with leukopenia instead of leukocytosis. Elevated liver enzymes are appreciated in 70% of cases of DRESS syndrome and will persist for several days after stopping the offending agent [4].

Clozapine is an atypical antipsychotic that is used to treat schizophrenia that is refractory to other psychiatric medications. Common side effects of clozapine include hepatocellular hepatitis, liver enzyme elevations, pancreatitis, hyperglycemia, eosinophilia, and pleural effusion [5]. The incidence of eosinophilia has been reported to be between 0.2% and 62% and it will usually present between weeks 3–5 of initiating clozapine. Elevated liver enzymes have also been seen with clozapine use and are seen in 30–50% of cases. Liver enzyme elevation will usually present between 4 and 5 weeks of initiating clozapine [6]. Thus far in the literature, only one case of DRESS syndrome secondary to clozapine exposure has been shown [3]. This case describes a typical case of DRESS syndrome secondary to clozapine initiation.

## 2. Case Description

A 32-year-old female with a past medical history significant for schizophrenia, bipolar disorder, and hepatitis C antibody positive presented from the behavioral health center for a 2-day history of a diffuse rash. The rash had started on her upper extremities and then spread to her face, chest, and thighs 2 days prior to admission. At times, the rash had been itchy and the patient had reported chills. She had been started on clozapine 10 days prior to admission. On admission she was febrile to 38.1°C, tachycardic to 113 bpm, and hypotensive to 96/63. On exam, she had a maculopapular rash that was nonblanching over her entire body except her lower legs. There was no mucosal involvement, but she had mild facial edema. Pertinent admission labs included WBC 6.9 (3.7–11.4 10^3^/*µ*L), Hgb 10.6 (10.8–15.3 g/dL), platelet 196 (140–393 K/*µ*L), eosinophils 10 (0–6%), eosinophils absolute 0.7 (0.0–0.5 10^3^/*µ*L), aspartate aminotransferase 81 (14–36 U/L), alanine aminotransferase 125 (9–52 U/L), alkaline phosphatase 155 (38–126 U/L), and hepatitis C antibody positive. Her urinalysis showed moderate leukocyte esterase with white blood cells, squamous cells, and few bacteria. Imaging on admission included chest X-ray, which showed a small left sided pleural effusion. A CT chest was done which showed minimal bilateral atelectasis with trace pleural effusions and cholelithiasis with contracted gallbladder and pericholecystic fluid.

At this point, there was concern for infectious etiology, and blood cultures were drawn. The patient was then started on broad-spectrum antibiotics with cefepime and vancomycin. Clozapine was stopped and her benztropine and lithium were initially continued as she had been on these for many years. After being seen by psychiatry, they were also discontinued. She was given Benadryl for her rash. Hepatitis C viral PCR pathogens were found to be negative. Blood cultures grew staphylococcus coagulase negative and urine culture showed mixed flora with no infection. The blood cultures were thought to be a contaminant, so they were redrawn and the second set did not grow anything. Antibiotics were discontinued, as there was no further concern for infectious etiology. Eosinophils continued to be elevated and she developed a leukocytosis. The next three days, her white blood cell count continued to rise and peaked at 26.5 10^3^/*µ*L. She had worsening fever and became more tachycardic. Liver enzymes continued to rise, so an ultrasound abdomen was performed due to concern for cholecystitis, which was negative. Antibiotics were restarted due to concern for infection. Her eosinophil count rose and peaked at 14%. She had an echocardiogram to rule out endocarditis, which was within normal limits. CT abdomen and pelvis with contrast was done to rule out an abdominal infection. It showed colon thickening, cholelithiasis, trace pleural effusions, and porta hepatis lymphadenopathy. Antibiotics were all discontinued again as infectious etiologies had been ruled out. At this point, there was concern for drug reaction with eosinophilia and systemic symptoms (DRESS syndrome) as she had the classic signs of DRESS including eosinophilia, skin rash, lymphadenopathy, and elevated liver enzymes. Other viral exanthema reactivations that are associated with DRESS were also considered. EBV, HHV-6, and HHV-7 were drawn, and all came back negative. Mononucleosis testing was not done on presentation although this can present as DRESS syndrome.

Dermatology was also consulted and agreed that this was DRESS syndrome. Methylprednisone IV 125 mg three times daily was started along with clobetasol twice a day. After starting prednisone, fever and white blood cell count improved. Eosinophils trended back down to normal limits and liver enzymes started trending down. She was discharged on an oral prednisone taper for 12 days. Outpatient follow-up did not show any further eosinophilia, white blood cell elevation, or elevated liver enzymes. Rash improved and disintegrated.

## 3. Discussion

This case demonstrates a typical case of DRESS syndrome with rash, lymphadenopathy, eosinophilia, and multiorgan involvement. This is the second reported case in the literature of clozapine-induced DRESS syndrome. DRESS syndrome has not been reported as a common side effect. Only one other case in the literature reports DRESS syndrome secondary to clozapine, and that case presented with no rash or lymphadenopathy and only multiorgan involvement and eosinophilia [3].

This is the first case to present with the common manifestations of DRESS syndrome including eosinophilia, rash, lymphadenopathy, and liver involvement after clozapine use.

A literature review of 172 cases of DRESS syndrome demonstrated that the average time of onset of DRESS syndrome after initiating the offending drug was 3.9 weeks (SD was 2.3) [2]. This case demonstrates an earlier onset of DRESS syndrome after just 1.5 weeks of starting clozapine. Many cases of DRESS syndrome have been reported after only 1–1.5 weeks of starting the offending drug. It is evident that this was still DRESS syndrome as the patient improved after treatment for DRESS syndrome was initiated which included cessation of clozapine, corticosteroids, and supportive care [7].

The patient had been on benztropine, lithium, and clozapine when she presented to us. She had been on benztropine and lithium for many years due to her extensive psychiatric history. Lithium can also cause DRESS syndrome, but is less likely in this case as she had been on it for so many years. Clozapine was the only new drug that she had been started on and it had been started 10 days prior to admission. Prior to starting clozapine, she had not had eosinophilia, so it is likely that this was due to clozapine-induced DRESS syndrome.

Eosinophilia has been reported as a common side effect of clozapine. Usually, it will present at least 3 weeks after starting clozapine [6]. In this case, the eosinophils began to rise 3 days after initiating clozapine. Further, this case points to DRESS syndrome rather than isolated eosinophilia as a side effect of clozapine as the patient presented with the other manifestations of DRESS syndrome.

## Data Availability

All data involved in this case report are described above. The articles cited to come to the conclusions can be found in the references section. All articles were accessed through the PubMed database.
